# Pregnancy-related death rates by county, race, and ethnicity in the US, 2000–2019

**DOI:** 10.1186/s13690-025-01688-z

**Published:** 2025-11-13

**Authors:** Rachel A. Zajdel, Erik J. Rodriquez, Paula D. Strassle, George A. Mensah, Gina S. Wei, Laverne G. Mensah, Parkes Kendrick, Kelly Compton, Yekaterina O. Kelly, Mathew M. Baumann, Zhuochen Li, Dillon O. Sylte, M. Ashworth Dirac, Christopher JL Murray, Laura Dwyer-Lindgren, Ali Mokdad, Eliseo J. Pérez-Stable

**Affiliations:** 1 Division of Intramural Research, National Heart, Lung, and Blood Institute, Bethesda, MD USA; 2 Division of Intramural Research, National Institute On Minority Health and Health Disparities, Bethesda, MD USA; 3 Center for Translation Research and Implementation Science, National Heart, Lung, and Blood Institute, Bethesda, MD USA; 4 Division of Cardiovascular Sciences, National Heart, Lung, and Blood Institute, Bethesda, MD USA; 5 Division of Intramural Research, Eunice Kennedy Shriver National Institute of Child Health and Human Development, Bethesda, MD USA; 6 Office of the Director, National Institute On Minority Health and Health Disparities, Bethesda, MD USA; 7 Institute for Health Metrics and Evaluation, University of Washington, Seattle, Washington, USA; 8 Department of Family Medicine, University of Washington, Seattle, Washington, USA; 9 Department of Health Metrics Sciences, University of Washington, Seattle, Washington, USA

**Keywords:** County-level, Health disparities, Maternal mortality, Pregnancy-related deaths, Race and ethnicity

## Abstract

**Background:**

Substantial disparities across geographies and racial and/or ethnic populations contribute to increasing maternal mortality in the US. The present study adds to existing literature by examining pregnancy-related death rates by race and/or ethnicity at the county level and over time.

**Methods:**

We used data from the US National Vital Statistics System and the US National Center for Health Statistics to estimate age-standardized pregnancy-related death rates as maternal deaths per 100,000 females aged 15 to 44 years from 2000 to 2019. We utilized validated small-area estimation methods and adjusted pregnancy-related death rates to account for misreporting of race and/or ethnicity on death certificates. Estimates were stratified by US county and racial and/or ethnic population (American Indian or Alaska Native [AIAN], Asian, Black, Latina, and White).

**Results:**

Pregnancy-related death rates increased for all racial and/or ethnic populations from 2000 to 2019, and in 99.4% (3062 of 3079) of counties (87.4% statistically significant). White women experienced the greatest median percent increase in pregnancy-related death across counties (214.7%) from 2000–2019, although they did not experience the highest burden in absolute terms. In 2019, the highest pregnancy-related death rates were observed among AIAN and Black women (3.5 and 3.7 deaths per 100,000) and in counties in the Northern Great Plains and Southern regions. Asian and Latina women experienced relatively lower pregnancy-related death rates (1.0 and 1.3 deaths per 100,000), yet they also exhibited an increase in pregnancy-related death since 2000.

**Conclusions:**

Pregnancy-related death rates continued to rise in the US through 2019 and disparities by race and/or ethnicity and geography remain stark. Clinical and public health interventions are urgently needed to address disparities and decrease maternal mortality.

**Supplementary Information:**

The online version contains supplementary material available at 10.1186/s13690-025-01688-z.

**Table:** 

Text box 1. Contributions to the literature
• The present study adds a unique county-level analysis of the geographical variation and racial and/or ethnic disparities in pregnancy-related deaths to existing literature.
• The highest pregnancy-related death rates were observed among American Indian and Alaska Native and Black women and in counties in the Northern Great Plains and Southern regions.
• This study provides evidence of demographic disparities in pregnancy-related death rates, which can inform county, state, and federal health officials where interventions are most needed.

## Background

Over 1200 women died due to maternal causes in the US in 2021, and this number has risen over the years [1]. The US maternal mortality ratio, measured as the number of maternal deaths per 100,000 live births [2], was 20.1 deaths in 2019 and rose to 32.9 deaths in 2021 [1], before decreasing in 2022 to 22.3 deaths [3], which is triple that of other high-income countries [4]. The high burden of maternal mortality in the US signifies a critical public health problem that needs addressing.

Although there are multiple ways to assess maternal mortality (appendix p 63), the present study uses a definition that most closely aligns with the World Health Organization’s (WHO) and Centers for Disease Control and Prevention’s (CDC) term “pregnancy-related deaths” [2, 5]. Pregnancy-related deaths encompass deaths occurring during pregnancy, delivery, and up to one year postpartum [6]. The majority of these deaths take place in the postpartum period (65%), whereas about 22% happen during pregnancy and 13% during the day of delivery [6]. Hemorrhage, cardiac conditions, infection, and thrombotic embolism constitute the leading causes of maternal mortality [6], although such estimates generally exclude suicide and drug overdose. A recent study determined that 84% of pregnancy-related deaths in 2017–2019 could have been averted by one or more reasonable changes to patient, community, clinician, facility, and/or systems factors [6]. This opportunity for prevention of death makes it a priority area for intervention.

Racial and/or ethnic and geographical disparities in US maternal mortality are long-standing. In 2021, the maternal mortality ratio among Black women (69.9 deaths per 100,000 live births) was 2.6 times higher than that of White women (26.6 per 100,000 live births) and similar disparities were observed for American Indian and Alaska Native (AIAN) women [1, 7]. Geographical disparities in maternal mortality are also prominent in the US, with the South and rural areas exhibiting significantly higher burden than other regions and metropolitan areas, respectively [8, 9].

Few studies have examined maternal mortality nationwide and none have examined maternal mortality at the intersection of race and/or ethnicity and county of residence. A recent study examined maternal mortality ratios from 1999 to 2019 at the state level and found that the highest increases in maternal mortality for all populations generally took place in states in the Midwest (Illinois, Indiana, Kansas, Missouri) and South (Georgia, Louisiana, Tennessee) census regions [10]. The present study adds to existing literature by assessing trends in pregnancy-related death rates by race and/or ethnicity at the county level.

## Methods

This analysis used methods previously developed for estimating cause-specific mortality by county and racial and/or ethnic population [11, 12]. We provide a summary of these methods as applied to maternal mortality below.

### Data

We used de-identified death records from the US National Vital Statistics System and population estimates from the US National Center for Health Statistics (NCHS) from 2000 to 2019 for this analysis (appendix p 32). We tabulated these data by county, age group (5-year age bands from 10–14 to 50–54), racial and ethnic population, and year. Some county boundaries changed over the analysis period; therefore, we used a previously developed mapping of counties to temporally stable geographical units [13], which reduced the number of areas analyzed from 3143 to 3110 counties or combined county units (appendix pp 30–31).

Race and/or ethnicity were combined into a single categorization with five mutually exclusive populations: AIAN, Asian or Pacific Islander (Asian), Black, Latina or Hispanic of any race (Latina), and White. Due to constraints in the underlying data (appendix p 4), we combined the Asian and Native Hawaiian or Pacific Islander (NHPI) populations for this analysis; we refer to this combined population as Asian. For death certificates where the individual was identified as having multiple racial identities, we used the primary (or bridged) race imputed by the NCHS [14].

We used the cause list and hierarchy developed for the Global Burden of Diseases, Injuries, and Risk Factors (GBD) 2021 study [15], and the associated mapping of ICD-10 codes [16] to GBD causes (appendix pp 33–48). For this analysis of maternal mortality, we included deaths among females ages 10–55 years and ICD-10 codes C58–C58.0, N96, N98–N98.9, O00–O07.9, O08*–O08.9^*^, O09–O16.9, O17^*^–O19^*^, O20–O26.9, O27^*^, O28–O36.9, O37^*^–O39^*^, O40–O48.1, O49^*^–O59^*^, O60–O77.9, O78^*^–O79^*^, O80–O92.7, O93^*^–O95.9^*^, O96–O98.6, and O98.8–O99.9. This definition includes late maternal deaths (those occurring more than 42 days but within 1 year of pregnancy) and is different from the CDC definition for maternal mortality, which only includes deaths occurring within 42 days of birth; instead, it is similar to how the CDC defines pregnancy-related deaths [5]. Although this makes comparison of absolute numbers somewhat difficult, the relative trends and differences by county and race and/or ethnicity are similar [17]. Importantly, this definition of maternal mortality does not include deaths due to suicide, substance use overdose, or homicide, as is consistent with the WHO’s definition of maternal mortality [18]. We also applied algorithms developed for the GBD study to reassign codes (marked with an asterisk above) assigned as an underlying cause of death that refer to an intermediate or immediate cause of death, are otherwise implausible, or are insufficiently specific to the likely true underlying causes of death (appendix pp 5–6) [15, 19]. All causes in the GBD cause list with at least 10,000 deaths in total over the study period and at least 1000 deaths each among males and females separately were analyzed concurrently.

Data on income and population density by county, and data on post-secondary education, poverty, and birthplace (in the US vs outside the US) by county and race and/or ethnicity, extracted from various sources, were also incorporated as covariates in the statistical model to better inform the estimates (appendix pp 6–9, 49–51). We used published estimates of race and/or ethnicity misclassification ratios, defined as the ratio of deaths among individuals of a particular population as indicated by self-report to deaths among individuals of that same racial and/or ethnic population as indicated on death certificates [20].

This study complies with the Guidelines for Accurate and Transparent Health Estimates Reporting (appendix p 3) [21]. This research received institutional review board approval from the University of Washington. No primary data were collected for this study, and we had no contact with human subjects.

### Statistical analysis

The statistical analysis was done in three stages. First, we used small-area estimation models to estimate pregnancy-related death rates by county, racial and ethnic population, age, and year, using the racial and ethnic identity reported on death certificates. The purpose of these models is to estimate the underlying mortality rate while smoothing out stochastic noise. Models were fit using the Template Model Builder package [22] in R version 3.6.1, and 1000 draws of the mortality rate were simulated from the approximated posterior distribution after fitting the models. Further details on model specification, model validation, and model performance are provided in the appendix (pp 9–22). Second, we used race and ethnicity misclassification ratios to adjust draws of the mortality rate derived from the small-area model (appendix pp 22–24). Third, to guarantee consistency among causes and that adjustment for misclassification did not change the overall mortality rate estimated for a given county, we did a post-hoc calibration using a two-stage iterative proportional fitting algorithm (appendix pp 24–27).

Final point estimates were derived from the mean of the 1000 draws, and 95% uncertainty intervals (UIs) were derived from their 2.5th and 97.5th percentiles. We generated estimates at aggregate geographical levels (ie, state and national) by population-weighting the age-specific mortality rates and calculated age-standardized mortality rates for ages 15–44 using the population distribution from the 2010 Census. When comparing any pair of age-standardized mortality estimates, we describe the difference as statistically significant when the posterior probability that the difference is greater than 0 is less than 2.5% or greater than 97.5%, akin to a two-tailed test with $\alpha$ = 0.05. We are using the term “statistically significant” and the threshold of 0.05 outside of the hypothesis testing context to help succinctly reflect the degree of uncertainty in the estimates, as this can vary widely by county and racial and ethnic population. However, we note that a statistically significant difference is not equivalent to a meaningful difference from a population health perspective; careful consideration of each estimate and its associated 95% UI is required to determine if a difference is meaningful in any given context.

While all available data were included in the statistical analysis, we masked (did not display) the modeled mortality rates in every year for county and racial and ethnic population combinations that had a mean annual population (for all sexes combined) of less than 1000 because model performance declined below this threshold (appendix pp 18–22). Thus, we report estimates for 3079 (of 3110) counties for the total population, 474 for the AIAN population, 667 for the Asian population, 1488 for the Black population, 1478 for the Latina population, and 3051 for the White population. Over 97% of the population in each racial and ethnic population other than AIAN lived in the counties with unmasked estimates; 82% of the AIAN population lived in counties with unmasked estimates (appendix pp 52–53).

## Results

**Fig. 1:**
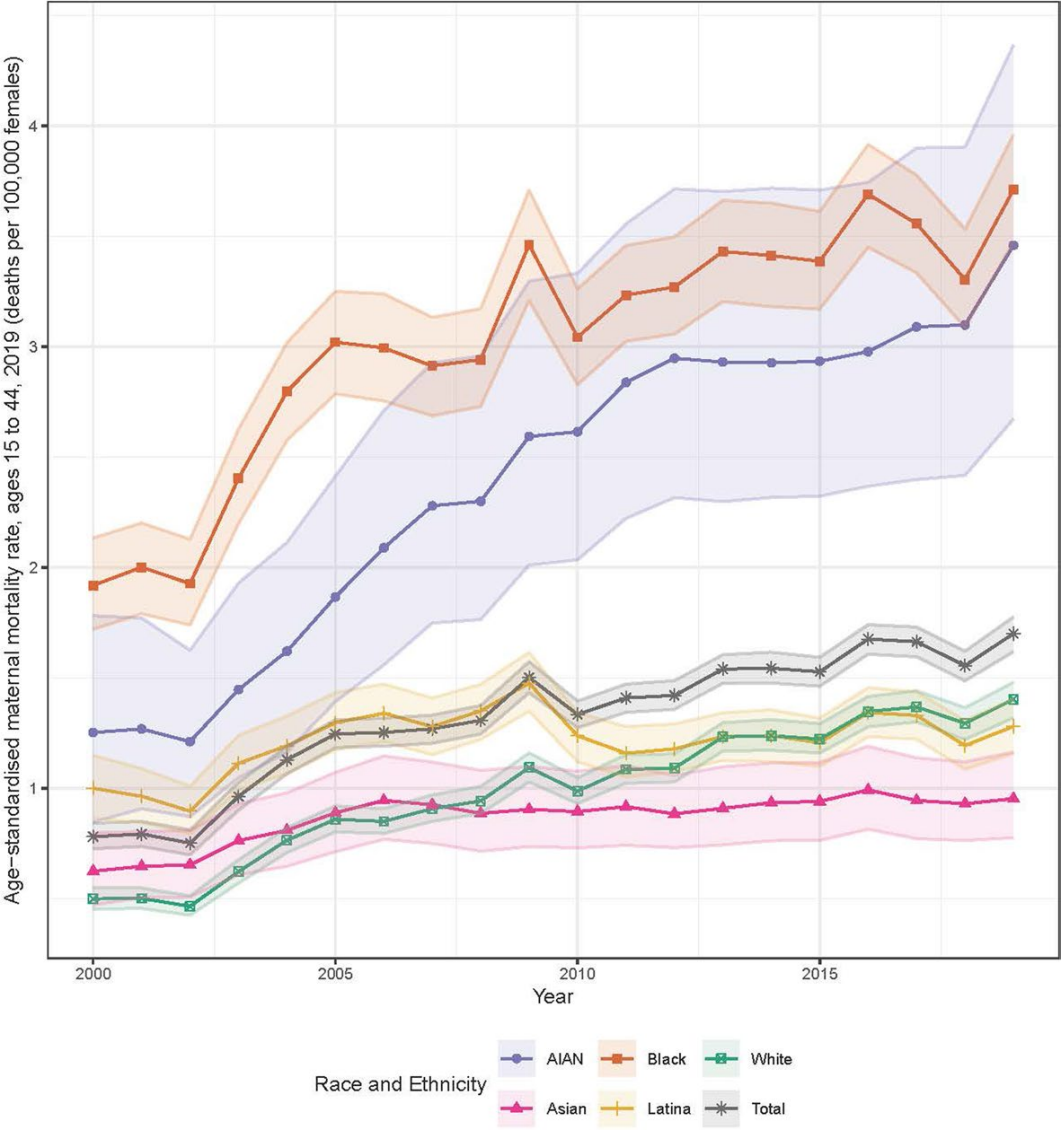
National-level, age-standardized pregnancy-related death rates (ages 15–44) per 100,000, from 2000 to 2019, by race and/or ethnicity. Shaded areas indicate 95% uncertainty intervals

Age-standardized pregnancy-related death rates among women of reproductive age (15–44 years) increased for all racial and/or ethnic populations, from 0.8 deaths per 100,000 females (95% UI 0.7–0.8) in 2000 to 1.7 per 100,000 (1.6–1.8) in 2019 (Fig. 1). Black women experienced the highest pregnancy-related death rates throughout the study period, from 1.9 deaths per 100,000 females (1.7–2.1) in 2000 to 3.7 per 100,000 (3.5–4.0) in 2019. AIAN and White women also experienced large, statistically significant increases in pregnancy-related death from 2000 to 2019, from 1.3 deaths per 100,000 females (0.8–1.8) to 3.5 per 100,000 (2.7–4.4) for AIAN women and 0.5 per 100,000 (0.5–0.5) to 1.4 per 100,000 (1.3–1.5) for White women. In contrast, Asian and Latina women had modest increases in pregnancy-related death over this period, from 0.6 deaths per 100,000 females (0.5–0.8) to 1.0 per 100,000 (0.8–1.2) among Asian women and from 1.0 per 100,000 (0.9–1.1) to 1.3 per 100,000 (1.2–1.4) among Latina women. The White population had the lowest pregnancy-related death rate from 2000 to 2007, whereas the Asian population had the lowest rate from 2008 to 2019.

### County-level variation in pregnancy-related death by race and/or ethnicity

**Fig. 2:**
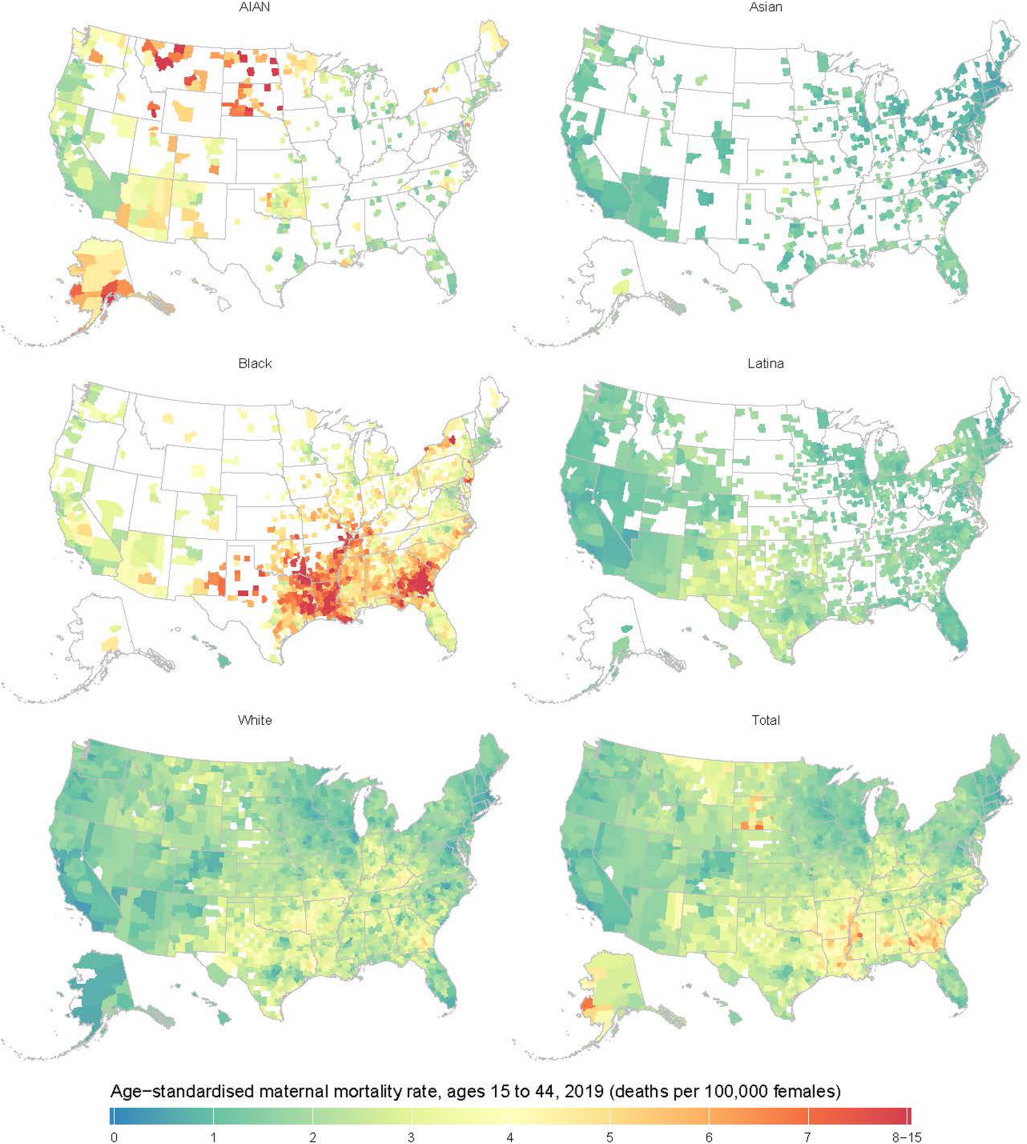
Estimated age-standardized pregnancy-related death rates (ages 15–44) per 100,000 at the county level in 2019, by race and/or ethnicity. Estimates have been masked (shown in white) for counties and racial and/or ethnic populations with a mean annual population fewer than 1000 people because model performance declined notably below this threshold

In 2019, differences in pregnancy-related death by race and/or ethnicity also existed at the county level (Fig. 2). AIAN women experienced the highest pregnancy-related death rates in counties in Alaska (although uncertainty intervals are large) and the Northern Great Plains states and comparatively lower rates in counties in California and Oregon. Of the ten counties with the highest pregnancy-related death rates among AIAN women in 2019, three were in Montana and three were in North Dakota. Among Black women, high pregnancy-related death rates clustered in counties in the South, whereas rates were lower in New England and Western counties. Nine of the ten counties with the highest pregnancy-related death rates among Black women in 2019 were in Georgia.

Fewer areas of high maternal mortality were observed for the Asian, Latina, and White populations. Of the ten counties with the highest pregnancy-related death rates for the Asian population, two were in Alaska and two were in Washington. Among Latina women, Texas held eight of the top ten counties with the highest pregnancy-related death rates. Five counties in Georgia were among the top ten counties for White women.

In the overall population, maternal mortality was highest in counties in the Northern Great Plains and the South, primarily driven by high rates of pregnancy-related death among AIAN women in Montana, North Dakota, and South Dakota, and high rates among Black women in Georgia, Louisiana, and Eastern Texas. Five of the ten counties with the highest pregnancy-related death rates in the overall population in 2019 were in Georgia.

### County-level changes in pregnancy-related death from 2000–2019

**Fig. 3:**
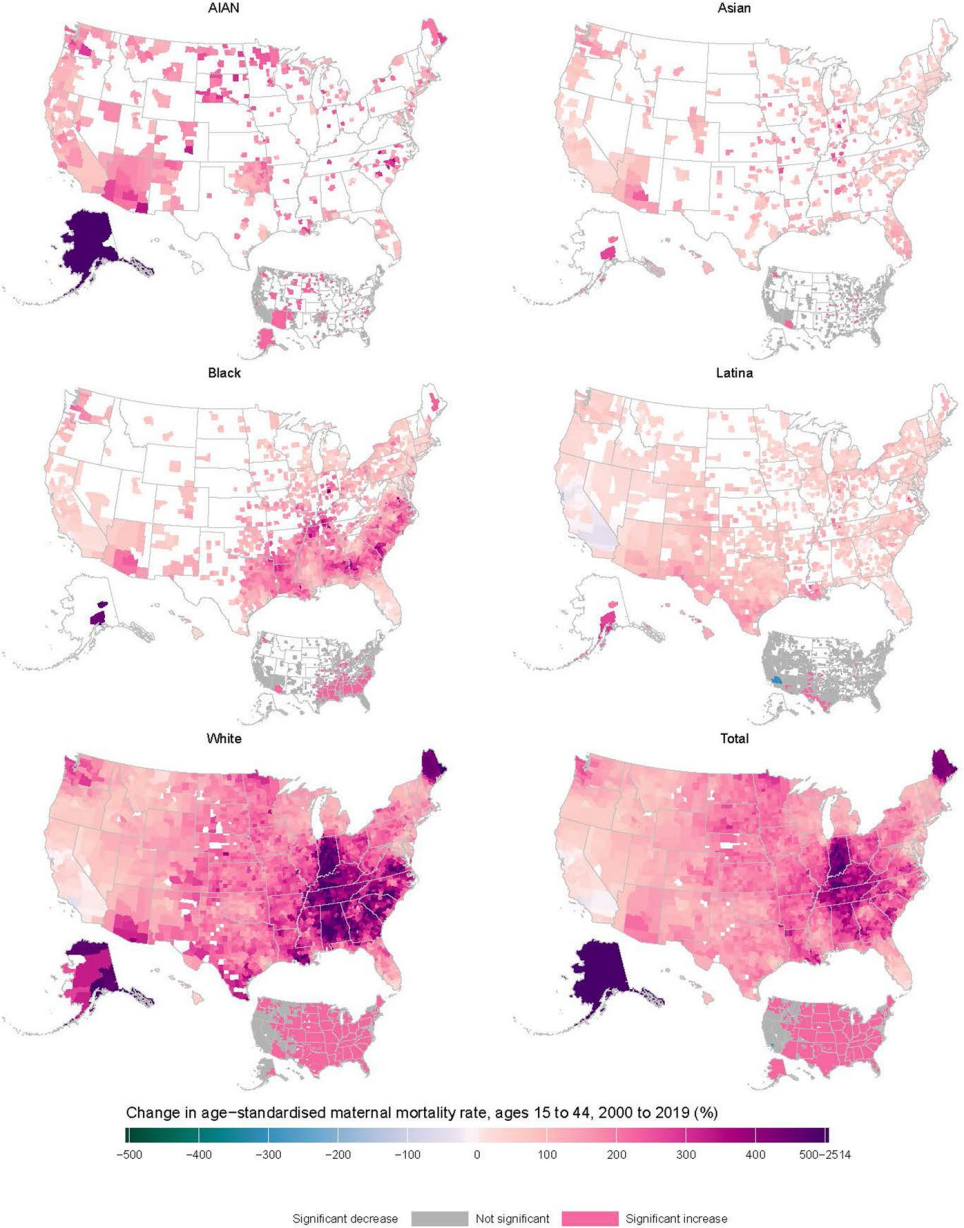
Percentage change in age-standardized pregnancy-related death rates (ages 15–44) between 2000 and 2019, by county and race and/or ethnicity. Estimates have been masked (shown in white) for counties and racial and/or ethnic populations with a mean annual population fewer than 1000 people because model performance declined notably below this threshold. Inset maps show where the change in mortality was statistically significant

Pregnancy-related death has been increasing at the county level across the US, with 99.4% (3062 of 3079) of counties with unmasked estimates experiencing a rise in pregnancy-related deaths between 2000 and 2019, and 87.4% statistically significantly so (Fig. 3). In the overall population, the largest increases in pregnancy-related death were generally seen in counties in the Midwestern and Southern states, Alaska, and Maine. The ten counties with the largest increase in pregnancy-related death were all in Alaska. Of the 17 counties that experienced a decrease in pregnancy-related death between 2000 and 2019, only one was statistically significant (Los Angeles County, California).

Increases in pregnancy-related death rates were also observed in all counties for AIAN women (474 of 474 counties, 26.8% [127] significant), 99.4% of counties among White women (3033 of 3051 counties, 86.0% [2623] significant), and 98.6% of counties for Black women (1467 of 1488 counties, 39.9% [593] significant). Although Asian and Latina women also saw a rise in pregnancy-related death rates in most counties (98.7% [658 of 667] and 95.3% [1409 of 1478], respectively), few of these counties had statistically significant changes (5.2% [35 of 667] and 3.7% [54 of 1478] significant for Asian and Latina women, respectively).

The median percent change in pregnancy-related death rates over time (2000–2019) was highest for White women, at 214.7% (IQR 160.4–312.0) among counties with unmasked estimates. White women experienced notable increases in pregnancy-related death in counties in the Midwest, South, Alaska, and Maine. For AIAN women, county-level changes in pregnancy-related death rates had a median value of 149.8% (IQR 109.2–194.8), with the greatest increases in Alaskan and Western counties. For the Black population, the median increase in pregnancy-related death was 129.2% (IQR 79.6–187.0) and the areas of notable increase were mainly found in the South census region and Alaska. The Asian population exhibited a 74.4% median increase in pregnancy-related death rates (IQR 46.5–107.7), with the greatest increases in counties in Alaska, Arizona, and the Midwest census region. Meanwhile, the Latina population saw the smallest median increase in mortality of 57.4% (IQR 34.0–84.0), with the largest increases in counties in the Southwest and Alaska.

## Discussion

The excess maternal mortality in the US is a national crisis that demands the attention of clinicians and public health scientists. This analysis showed that the pregnancy-related death rate has increased for all racial and/or ethnic populations in most counties through 2019. The unique county-level analysis indicates geographical variation and racial and/or ethnic disparities in the burden of pregnancy-related death that may inform where interventions are most needed.

Although AIAN and Black women experienced the highest burden of pregnancy-related death in 2019, and White women experienced the greatest increase from 2000 to 2019, Asian and Latina women also had higher death rates than populations in other high-income countries. For example, AIAN, Black, and Latina women had higher pregnancy-related death rates in 2019 in every county with unmasked estimates than the overall rate in Canada (0.44 deaths per 100,000 women ages 15–44)[23, 24] and Asian and White women had higher pregnancy-related death rates than Canada in more than 99% of US counties. As this comparison demonstrates, maternal mortality in the US is unacceptably high for all populations.

A notable spatial pattern from the present study is the concentration of higher rates of pregnancy-related death among Black women in 2019 in the Southern Mississippi basin, Eastern Texas and South Georgia. Another pattern is the substantial increases in pregnancy-related death among White women in many counties in the Midwest, South, Alaska, and Maine. A previous study found that maternal mortality was associated with socioeconomic and health care access factors at the county level [25]. Nevertheless, it is not clear why pregnancy-related death was so high during this time period, in these specific geographical clusters, compared with other counties which are also affected by similar factors that contribute to maternal mortality. Uncovering the reasons behind these spatial trends is a critical area for future research.

Factors that contribute to maternal mortality disparities include access and quality of health care before, during, and after pregnancy; unequal exposure to chronic stressors; intra-hospital care during the delivery; and complications exacerbated by a high prevalence of chronic conditions [4, 26]. Racial and/or ethnic minoritized women have less adequate health insurance and report experiences of racism, discrimination, and mistreatment in health care settings [27], whereas rural residents experience more shortages of specialized clinicians and greater distances to maternity care [28]. Delayed or lack of prenatal care is also associated with heightened risk of pregnancy-related death [29]. Restrictive reproductive health policies may also exacerbate these disparities [30, 31], and thus, access to and quality of primary and reproductive care may explain some of the observed disparities.

Interventions have been proposed to address health system failings in maternal care, such as training in implicit bias and respectful maternity care, standardization of protocols, use of patient checklists, implementation of triggers to mandate further action, and use of synergistic sets of interventions performed together [27, 32]. Furthermore, to address the maternal health of women in non-metropolitan areas, effective initiatives have been enacted to increase the availability of affordable maternal health services and the number of obstetricians and clinicians with rural training experience [33, 34].

Given that the majority of maternal deaths occur postpartum [4, 6], maternal health care must be addressed in the year following delivery [35]. Women need to have access to high-quality primary care to evaluate symptoms, appropriately diagnose problems, and avoid the consequences of newly developed complications such as cardiovascular events and, although not captured in the present analysis, postpartum depression. However, even when access to care is available, individuals from minoritized racial and ethnic populations experience more barriers that impede early detection, proper diagnosis, adequate treatment, access to contraception, and attendance at appointments in the postpartum period [36, 37]. Expanding health insurance coverage to one year postpartum [38] and implementing paid parental leave [39] have been found to improve maternal health outcomes. The postpartum year is therefore a period of high risk that warrants greater emphasis in primary care.

There is a need for more research on maternal health, given that the drivers of the rising rates of maternal mortality in the US are not well understood, especially in the postpartum period [40, 41]. The National Institutes of Health’s Implementing a Maternal Health and Pregnancy Outcomes Vision for Everyone (IMPROVE) initiative, which was launched in 2019 to support research that addresses preventable causes of maternal deaths and to improve health before, during, and after delivery, has started this process [42]. Other NIH efforts to advance maternal health and close the maternal health disparity gap are ongoing [43–45]. Lowering maternal mortality and coexisting disparities will require interdisciplinary research to advance knowledge and address the complicated system of factors that contribute to maternal mortality [40].

One potential confounder in evaluating the rise in pregnancy-related death rates is the introduction of pregnancy checkboxes to death certificates in many states, as this change allowed more deaths occurring to pregnant individuals to be ascertained [46]. The staggered implementation of the pregnancy checkboxes at varying times across states between 2003–2017 could also be impacting the observed spatial trends in pregnancy-related death. However, the introduction of pregnancy checkboxes does not entirely explain the observed increases in pregnancy-related death. By 2007, half of US states had adopted the pregnancy checkbox on death certificates [47]. A recent study found that maternal mortality ratios continued to increase for most states even after widespread adoption of the pregnancy checkbox [10]. Findings from the present study similarly show a sustained increase in pregnancy-related death rates for AIAN, Black, and White women even after 2007. From 2018 to 2019, after all states had implemented the change, pregnancy-related death rates continued to rise for all groups. Most importantly, the disparities by race and/or ethnicity observed in the present study cannot be explained by the introduction of pregnancy checkboxes. Even if the absolute values reported are higher than would be observed in the absence of pregnancy checkboxes on death certificates, AIAN and Black women would still be experiencing undue burden of pregnancy-related death relative to others.

It should also be noted that we were unable to capture changes in pregnancy-related death since 2019 reflecting effects of the COVID-19 pandemic. Maternal mortality initially rose during the pandemic, hitting a peak of 32.9 deaths per 100,000 live births in 2021 [3]. This increased mortality burden disproportionately affected Black and Latina women [48], paralleling trends in overall mortality by race and/or ethnicity observed during this period [49]. The U.S. Government Accountability Office estimated that COVID-19 contributed to 25% of maternal deaths in 2020 and 2021, as the pandemic exacerbated social vulnerabilities associated with maternal health, such as access to care [48]. Maternal mortality rates have since decreased to 18.6 deaths per 100,000 live births in 2023 [50]. It is unknown what lasting effects the pandemic may have on maternal mortality rates and disparities.

There are several other limitations of the present study. Estimates with large uncertainty intervals should be interpreted with caution [12]. Moreover, although the present analysis aligns with the WHO definition of maternal mortality, this does not include deaths related to mental health or substance use disorders, which is the leading cause of mortality in this age group among Latina and White women [6]. It is unclear what disparities the current study may reveal if these causes were included in the operationalization of maternal mortality. Although this study focused on pregnancy-related deaths, this alone does not convey the full scale of maternal ill-health in the US. For every individual who dies due to a pregnancy-related cause, up to 100 others experience severe maternal morbidity [51]. Lastly, the focus on broad racial and/or ethnic categories may have hidden critical disparities within each population such as by Latina [52] and Asian and NHPI [53] heritage. Despite these limitations, the present study provides a systematic analysis of pregnancy-related death rates across race and/or ethnicity, time, and geography and thus highlights enduring and emerging disparities in need of targeted policies and interventions.

## Conclusions

Pregnancy-related death rates rose in 2000–2019 in the US for all racial and/or ethnic populations in nearly all counties, and disproportionately impacted AIAN and Black women. These findings call for urgent implementation strategies that help accelerate the adoption and sustained use of proven-effective interventions for addressing preventable causes of maternal deaths. It is imperative to continue to identify upstream causes of maternal mortality while also testing and implementing effective strategies to eliminate persistent disparities and stop this crisis.

## Supplementary Information

Supplementary Material 1

## Data Availability

Estimates of pregnancy-related death by county, racial and ethnic population, year, age, and sex are available for download from the Global Health Data Exchange (link available upon publication) and via a user-friendly data visualization: https://vizhub.healthdata.org/subnational/usa. Information about the underlying data sources is available in the Supplementary Material (pp 32, 48–49). The code used for this analysis is available on GitHub: https://github.com/ihmeuw/USHD.

## Abbreviations

AIAN: American Indian or Alaska Native
CDC: Centers for Disease Control and Prevention
GBD: Global Burden of Diseases, Injuries, and Risk Factors
NHPI: Native Hawaiian or Pacific Islander
WHO: World Health Organization
